# Deleterious genetic changes in *AGTPBP1* result in teratozoospermia with sperm head and flagella defects

**DOI:** 10.1111/jcmm.18031

**Published:** 2023-11-08

**Authors:** Yu‐Hua Lin, Ya‐Yun Wang, Tsung‐Hsuan Lai, Jih‐Lung Teng, Chi‐Wei Lin, Chih‐Chun Ke, I‐Shing Yu, Hui‐Ling Lee, Chying‐Chyuan Chan, Chi‐Hua Tung, Donald F. Conrad, Moira K. O'Bryan, Ying‐Hung Lin

**Affiliations:** ^1^ Division of Urology, Department of Surgery Cardinal Tien Hospital New Taipei Taiwan; ^2^ Department of Chemistry Fu Jen Catholic University New Taipei City Taiwan; ^3^ Graduate Institute of Biomedical and Pharmaceutical Science, Fu Jen Catholic University New Taipei City Taiwan; ^4^ Department of Obstetrics and Gynecology Cathay General Hospital Taipei Taiwan; ^5^ School of Medicine, Fu Jen Catholic University New Taipei City Taiwan; ^6^ Department of Urology En Chu Kong Hospital New Taipei City Taiwan; ^7^ Laboratory Animal Center College of Medicine, National Taiwan University Taipei Taiwan; ^8^ Department of Obstetrics and Gynecology Taipei City Hospital, Zhongxing Branch and Branch for Women and Children Taipei Taiwan; ^9^ Program of Artificial Intelligence & Information Security Fu Jen Catholic University New Taipei City Taiwan; ^10^ Division of Genetics, Oregon National Primate Research Center Beaverton Oregon USA; ^11^ School of BioSciences and Bio21 Institute, The University of Melbourne Parkville Victoria Australia

**Keywords:** AGTPBP1, genetic changes, male infertility, teratozoospermia, whole‐exome sequencing

## Abstract

Approximately 10%–15% of couples worldwide are infertile, and male factors account for approximately half of these cases. Teratozoospermia is a major cause of male infertility. Although various mutations have been identified in teratozoospermia, these can vary among ethnic groups. In this study, we performed whole‐exome sequencing to identify genetic changes potentially causative of teratozoospermia. Out of seven genes identified, one, *ATP/GTP Binding Protein 1* (*AGTPBP1*), was characterized, and three missense changes were identified in two patients (Affected A: p.Glu423Asp and p.Pro631Leu; Affected B: p.Arg811His). In those two cases, severe sperm head and tail defects were observed. Moreover, AGTPBP1 localization showed a fragmented pattern compared to control participants, with specific localization in the neck and annulus regions. Using murine models, we found that AGTPBP1 is localized in the manchette structure, which is essential for sperm structure formation. Additionally, in *Agtpbp1*‐null mice, we observed sperm head and tail defects similar to those in sperm from AGTPBP1‐mutated cases, along with abnormal polyglutamylation tubulin and decreasing △−2 tubulin levels. In this study, we established a link between genetic changes in *AGTPBP1* and human teratozoospermia for the first time and identified the role of AGTPBP1 in deglutamination, which is crucial for sperm formation.

## 1 INTRODUCTION

Infertility has been recognized as a global public health concern by the World Health Organization (WHO), and affects at least 8%–12% of couples worldwide^1^, ^2^ and approximately 7% of all men.^3^, ^4^ Teratozoospermia is the leading clinical manifestation of male sterility, and is characterized by sperm head defects accompanied by sperm DNA damage.^5^ Teratozoospermia may affect the outcomes of assisted reproductive techniques,^6^ and is caused by mutations in several genes, including *AURKC*, *SPATA16*, *DPY19L2*, *DNAH1* and *SEPT12*.^7^, ^8^, ^9^

In the past decade, the identification of genetic variations (e.g., copy number variation, deletion, insertion, and nucleotide alterations) associated with male infertility using next‐generation sequencing (NGS) has increased.^8^ However, research on the aetiology of male infertility typically focuses on specific patient subgroups, such as men with teratozoospermia. NGS has been used to examine various subtypes of teratozoospermia, such as sperms with multiple flagellar morphological abnormalities,^10^ cases of primary ciliary dyskinesia‐associated male infertility,^11^ and globozoospermia.^12^ However, many plausible causes of male infertility have yet to be replicated and the aetiology may vary between populations.^8^

*AGTPBP1* is predominantly expressed in the testes and brain,^13^ and comprises an armadillo‐type fold and a carboxypeptidase A domain.^13^ The enzymatic functions of the carboxypeptidase A domain specifically remove polyglutamate from the C‐terminus of α‐tubulin and generate △−2 tubulin.^14^, ^15^, ^16^ Conversely, tubulin tyrosine ligase‐like family proteins (TTLL) add polyglutamate.^17^, ^18^ *AGTPBP1* mutations can cause childhood‐onset neurodegeneration with cerebellar atrophy (CONDCA) by decreasing deglutamylase activity and the protein amount of △−2 tubulin.^19^ Furthermore, deletion of the *Agtpbp1* allele in spontaneous strain mice not only causes neuronal degeneration but also results in male infertility.^20^, ^21^ AGTPBP1 regulates the post‐translational modifications of tubulin, such as polyglutamation, and generates △−2 tubulin. The development of male germ cells is highly reliant on the dynamics of microtubule structures, such as sperm head shaping and tail formation.^22^, ^23^ We suggest that mutated *AGTPBP1* may be involved in human teratozoospermia.

In this study, we screened patients with teratozoospermia in Taiwan for genetic alterations using whole‐exon sequencing (WES) and identified three genetic mutations in *AGTPBP1*. Notably, the sperm head and tail phenotypes observed in *Agtpbp1*‐defective mice resembled those observed in patients carrying *AGTPBP1* missense mutations, providing strong evidence that *AGTPBP1* is a human male infertility gene.

## 2 MATERIALS AND METHODS

### 2.1 Case enrolment

This study was approved by the Ethics Committee of Cathay General Hospital (IRB approval no: CGH‐P102031; CGHFJU‐105006). Informed consent was obtained from all recruited patients. Semen samples were obtained by masturbation after 3–5 days of sexual abstinence. After liquefying the semen, routine semen analysis was performed according to the WHO 2010 criteria.^24^ The results for the selected cases are listed in Table S1. Sperm morphology was evaluated using Kruger's criteria.

### 2.2 Genomic DNA extraction, WES and bioinformatic analysis

Genomic DNA was extracted from sperm using a QIAamp DNA Micro Kit according to the manufacturer's instructions and stored at −80°C until the experiment. DNA libraries were prepared using the Illumina TruSeq Exome Library Prep Kit and sequenced using the Illumina NextSeq platform. The average coverage depth was approximately 50×. Sequenced reads were mapped using the Burrows–Wheeler Aligner. WES reads were aligned to the human reference genome (Genome Reference Consortium Human Build 37). Small‐copy number variants, insertions, deletions, and variants were identified in individual cases. In collaboration with Professor Donald F. Conrad, the WES outputs were filtered and prioritized using the population sampling probability (PSAP) test, a statistical framework for assessing the significance of variants from single cases of rare genetic diseases.^25^ The WES data were filtered according to the following rules: (1) read depth > 10, (2) percentage of alternated sites of the reads/all reads >20%, and (3) PSAP <0.001. The selected variants were annotated using data from the Exome Aggregation Consortium (ExAC) genome database, and pathogenic effects were predicted using SIFT and PolyPhen‐2 softwares. Finally, the mutated sites of the candidate genes were confirmed by Sanger sequencing after polymerase chain reaction (PCR) using the reference transcript of *AGTPBP1* (NM_001286717.1). The following primes were used for the genomic typing of *AGTPBP1*: forward (F′: GGCTTCTGAGGGTTAATGG AG) and reverse (R′: TGGGGCTGAAGTAGGGTCTAA). To determine the effects of mutations within the protein structure, three‐dimensional (3D) models of mutated‐*AGTPBP1* were predicted using ColabFold^26^ and the carboxypeptidase domain as templates. The results were visualized using PyMOL software (PyMol Molecular Graphics System, Version 2.5.4).

### 2.3 Immunofluorescence assay (IFA) and transmission electron microscopy (TEM)

The AGTPBP1 localization profile in human testicular sections was obtained from The Human Protein Atlas (https://www.proteinatlas.org/ENSG00000135049‐AGTPBP1/tissue/testis#img).^27^ For IFA, slides were treated with 0.1% Triton X‐100, washed twice with Tris‐buffered saline (TBS), and incubated with the primary antibody (rabbit anti‐AGTPBP1 antibody, 1:1000 dilution, Proteintech, Cat. No.14067‐1‐AP; rabbit anti‐delta‐2 tubulin antibody, 1:500 dilution, Millipore, Cat. No.AB3203; mouse anti‐polyglutamylation modification antibody, 1:2000 dilution, AdipoGen, Cat. No.AG‐20B‐0020) at room temperature for 60 min. After washing with TBS, the sections were incubated with secondary antibody for 60 min at room temperature and washed again with TBS. An acrosome (lectin PNA, Thermo Fisher Scientific, Cat. No.L32458) and mitochondrial markers (Mito‐Tracker Red, Thermo Fisher, Cat. No.M7512) were used for the orientation. For TEM, spermatozoa were washed in 0.1 M phosphate buffer (pH 7.2), fixed and processed according to the protocol described in our previous study.^28^ The final sections were counterstained with lead citrate and uranyl acetate and observed using a JOEL 1200 TEM.^29^

### 2.4 Preparation of the murine testicular germ cell populations

All animal studies were approved by the Institutional Animal Care and Use Committee (No: A10871, date of approval: 04/10/2020; No: A10979, date of approval: 03/18/2021) of Fu Jen Catholic University. Murine male germ cells were isolated using a centrifugal system according to the density of different types of germ cells, as described previously.^28^ Briefly, testes were decapsulated and the seminiferous tubules were enzymatically digested, after which the germ cell suspensions were filtered through 35‐μM nylon filters (Falcon). The suspension of single cells was centrifuged at different gravity levels on a Kubota centrifuge 3330. Germ cells were collected at different developmental stages. Mature spermatozoa were collected from the cauda epididymides of adult male mice. Finally, suspended male germ cells were spread on a slide and air‐dried for further analysis.

### 2.5 Generation of *Agtpbp1* knockout mice using CRISPR/Cas9

sgRNA was designed by the Gene Knockout Mouse Core Laboratory of the National Taiwan University Center of Genomic Medicine to delete the critical functional carboxypeptidase domain of the *Agtpbp1* allele. The generated sgRNAs and Cas9 targeted the genomic *Agtpbp1* allele in C57BL/6 mouse embryonic stem cells (MESCs) to create a deletion (del) region **(**Figure 4A **)**. After replacing the wild‐type allele in MESCs, clones bearing the targeted allele were confirmed using PCR and sequencing. The confirmed clones were injected into C57BL/6J blastocysts. Blastocysts were transferred to pseudopregnant female mice. Male chimeras were mated with wild‐type females to generate *Agtpbp1* ^*+/del*^ mice. The reproductive ability of each group was compared among pups from the same pregnancy.

### 2.6 Sperm quality analysis

Spermatozoa collected from enrolled patients or the vas deferens of wild‐type (*n* = 5), *Agtpbp* ^*/+/del*^ (*n* = 5), and *Agtpbp* ^*del/del*^ (*n* = 6) adult male mice was suspended in human tubal fluid (HTF) medium (Irvine Scientific). To determine sperm counts, sperm were immobilized by dilution in water and counted using a haemocytometer in duplicate. To evaluate sperm morphology, the sperm medium was diluted to 10^6^/mL with HTF and spotted onto a glass slide. A total of 200 sperms (both motile and immotile) were counted under a microscope in duplicate to obtain the average percentage of motility.

### 2.7 Immunoblotting

Testicular tissues and sperms were homogenized using a tissue homogenizer in lysis buffer (20 mM Tris/HCl [pH 8], 150 mM NaCl, 5 mM MgCl_2_, 0.5% Triton‐X 100, 10% glycerol and a protease inhibitor cocktail). Total lysates were incubated for 30 min on ice and centrifuged at 10,000 × *g* and 4°C for 20 min. Protein extracts were heated for 5 min at 37°C before sodium dodecyl sulfate–polyacrylamide gel electrophoresis was performed on an 8% gel.^30^ The separated proteins were transferred onto polyvinylidene fluoride membranes and incubated with antibodies (rabbit anti‐AGTPBP1, Proteintech, Cat No. 14067‐1‐AP; rabbit anti‐delta‐2 tubulin, Millipore, Cat. No.AB3203; mouse anti‐polyglutamylation modification tubulin, AdipoGen, Cat. No.AG‐20B‐0020; mouse anti‐GAPDH antibody, Sigma‐Aldrich, Cat. No. G8795) and detected using a chemiluminescence detection system.

## 3 RESULTS

### 3.1 Identification of the novel genetic alterations in patients with teratozoospermia

**FIGURE 1:**
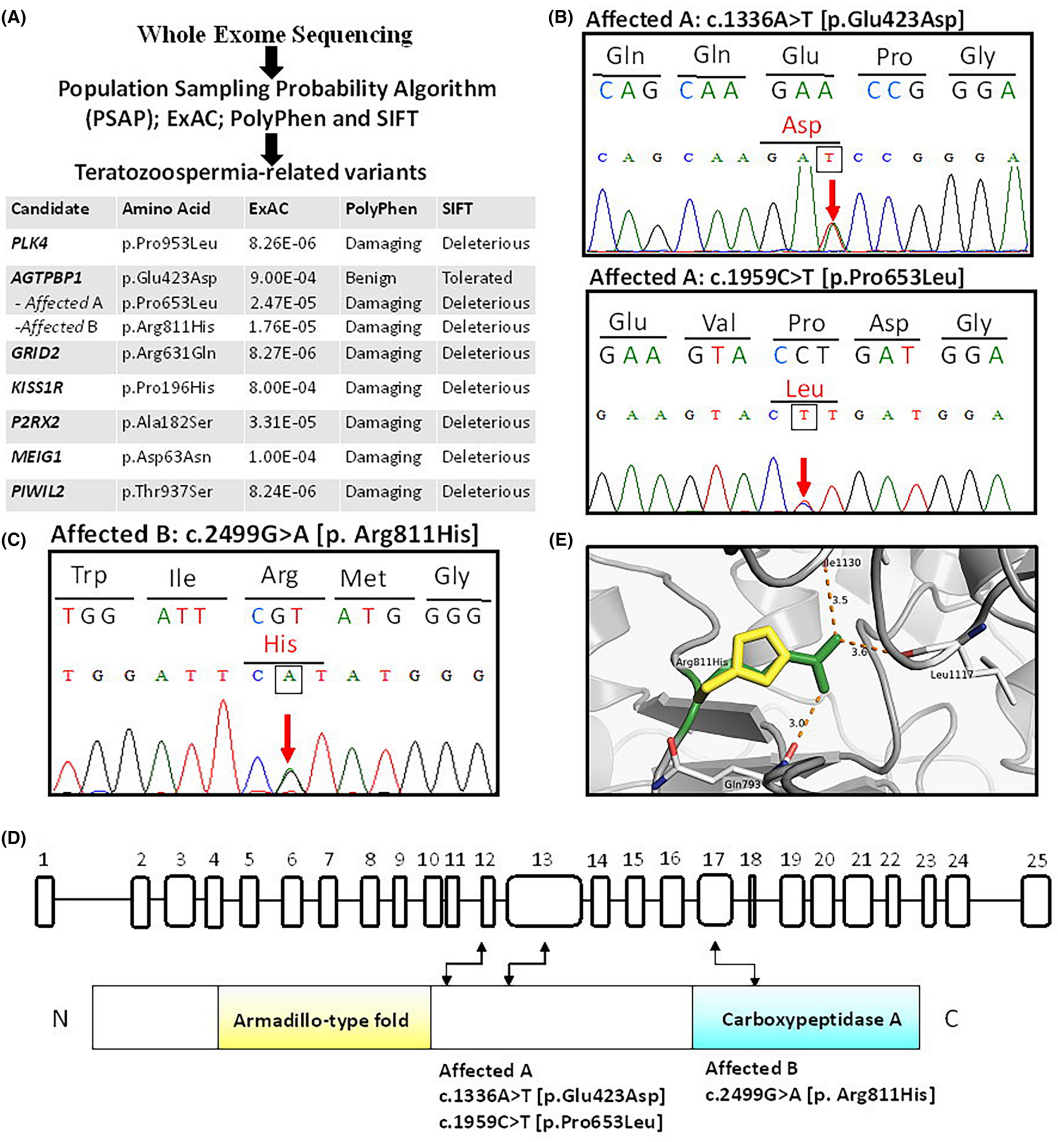
Identification of *ATP/GTP Binding Protein 1* (*AGTPBP1*) genetic alterations in teratozoospermia using whole‐exome sequencing (WES). (A) Screening for genetic changes in 12 teratozoospermia cases using WES and the results of bioinformatic analysis (PSAP; ExAC; PolyPhen, and SIFT). (B, C) Sanger sequencing chromatograms of *AGTPBP1* variants. Chromatograms display the sequence corresponding to the control and mutated alleles (upper and lower panels, respectively). Genetic changes in *AGTPBP1* in affected individual A carrying compound heterozygous mutations (NM_001286717.1: c.1336A > T [p.Glu423Asp]; c.1959C > T [p.Pro653Leu]) and affected individual B carrying a hemizygous mutation (NM_001286717.1: c.2499G > A [p. Arg811His]). Red arrows indicate the site changes compared with control cases. (D) Schematic of the *AGTPBP1* gene (upper panel) and protein (lower panel). The 25 exons of the human *AGTPBP1* were numbered. N = N‐terminal; C = C‐terminal. The near N‐terminal and C‐terminal regions of AGTPBP1 are encoded with the armadillo‐type fold and carboxypeptidase A, respectively. The amino acid of p.Arg811His was located in exon 17 of *AGTPBP1*. (E) The substitution of arginine (green colour) with histidine (yellow colour) (p.Arg811His) in the predicted carboxypeptidase A domain structure disrupts the oxygen–hydrogen bonding between the nearby amino acids (p.Gln793, Leu1117, and Ile1130).

To explore the genetic causes of teratozoospermia in Taiwan, we enrolled 254 males with infertility, defined as those with one or more abnormal semen parameters. Twelve patients with severe morphological sperm defects were selected (Table S1) and WES was performed. The identified genetic variants were evaluated using the PSAP test, allele frequencies from ExAC, and the pathogenicity predictors PolyPhen and SIFT (Figure 1A). Finally, heterozygous variants in seven teratozoospermia‐related genes were predicted to be potentially deleterious (Figure 1A: *PLK4*: p.Pro953Leu; *AGTPBP1*: p.Glu423Asp, p.Pro653Leu, p.Arg811His; *GRID2*: p.Arg631Gln; *KISS1R*: p.Pro196His; *P2RX2*: p.Ala182Ser; *MEIG1*: p.Asp63Asn; *PIWIL2*: p.Thr937Ser). One of the candidate genes, *AGTPBP1*, is remarkable, as a classical spontaneous *Agtpbp1* mouse mutant has been shown to exhibit abnormal sperm development in previous studies.^20^, ^21^ Genetic changes in *AGTPBP1* were observed in both cases. Affected individual A carried compound heterozygous mutations (NM_001286717.1: c.1336A > T [p.Glu423Asp] and c.1959C > T [p.Pro653Leu]) (Figure 1B). The other hemizygous mutation (NM_001286717.1: c.2499G > A [p. Arg811His]) was detected in affected individual B. (Figure 1C). Both PolyPhen and SIFT predicted that two amino acid changes (p.Pro653Leu and p.Arg811His) damaged the protein (Figure 1A). Furthermore, the changed site of p.Arg811His localized within the critical carboxypeptidase A domain of AGTPBP1 (Figures 1C,D). The predicted 3D protein structure revealed that the substitution of Arg811 with His would likely disrupt the oxygen–hydrogen bonding between nearby amino acids (p.Gln793, Leu1117, and Ile1130), potentially affecting structural stability and destabilizing the overall functional structure (Figure 1E). In summary, based on WES and bioinformatics tests, several genetic alterations were identified in patients with teratozoospermia, and *AGTPBBP1* genetic variations appeared to be associated with human teratozoospermia.

### 3.2 Spermatozoa from patients with altered *AGTPBP1* revealed severe morphological defects

**FIGURE 2:**
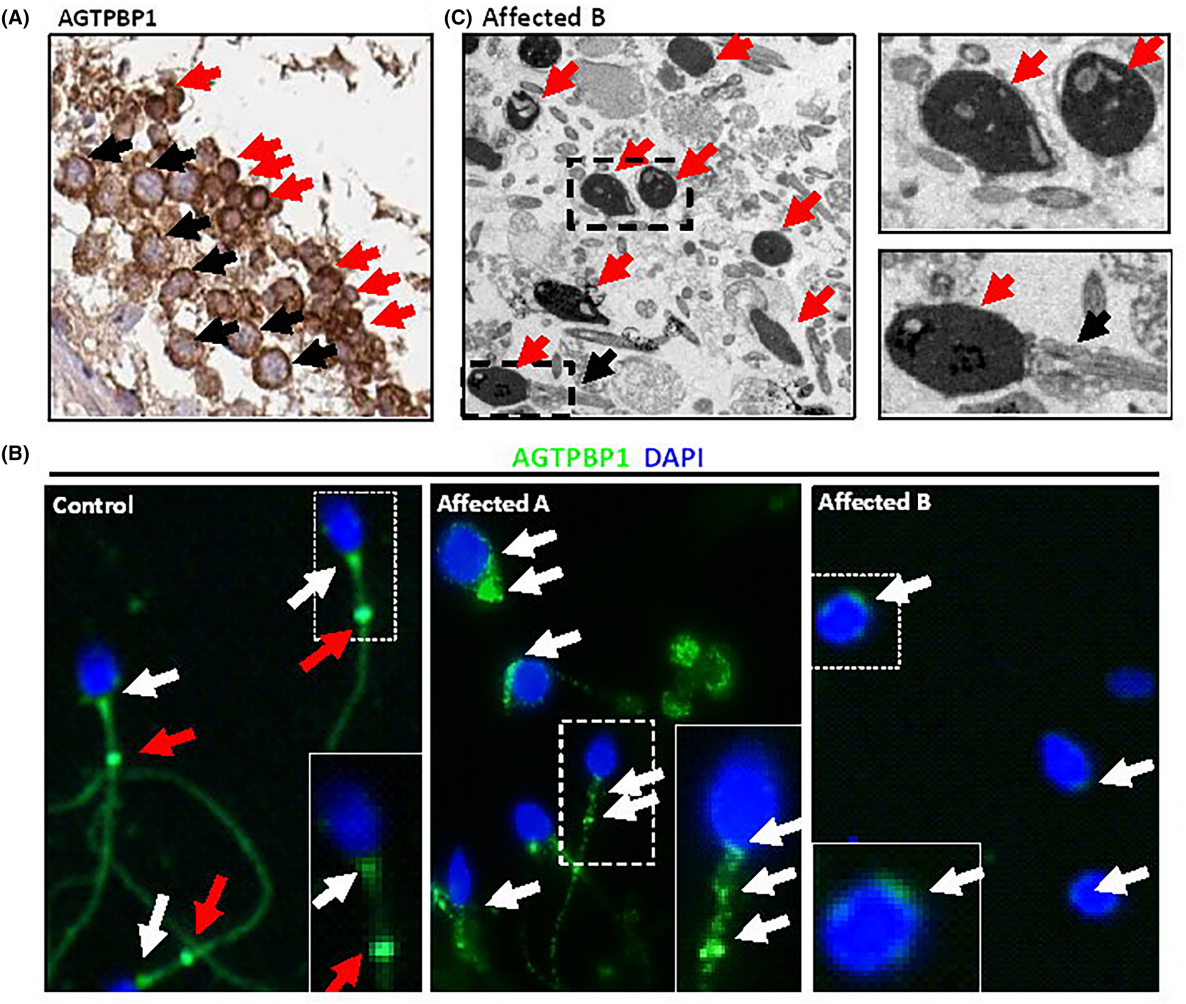
Immunostaining and electron microscopy analysis in cases with *ATP/GTP Binding Protein 1* (*AGTPBP1*) mutations. (A) Immunohistochemical detection of AGTPBP1 signals in the human testicular sections from The HUMAN PROTEIN ATLAS. Black and red arrows indicate the signals on spermatocytes and spermatids, respectively. (B) Sperm from the control (Left), affected individual A carrying compound heterozygous mutations (NM_001286717.1: c.1336A > T [p.Glu423Asp]; c.1959C > T [p.Pro653Leu]), and affected individual B carrying a hemizygous mutation (NM_001286717.1: c.2499G > A [p. Arg811His]). AGTPBP1 is mainly located at the sperm neck (white arrows) and annulus (red arrows) in control cases. AGTPBP1 signals (white arrows) denote the disrupted patterns (fragmented) (Middle and Left) and decreased AGTPBP1 signals (Left). Sperm head stains with nuclear dye (DAPI; blue). The sperm is marked in the dotted rectangle and enlarged in the right corner. (C) Electron microscopy images of sperm from affected individual B showing severely malformed head shapes (red arrow) and sperm tail defects (black arrow). Enlarge figures have shown as the right panels.

Immunostaining and TEM were used to examine genetic changes in *AGTPBP1* in spermatozoa. *AGTPBP1* was expressed in spermatocytes (black arrows) and was strongly expressed in spermatids (red arrows) in human testicular sections obtained from the Human Protein Atlas database (Figure 2A). Immunofluorescence staining of human spermatozoa revealed that *AGTPBP1* was mainly localized in the neck (white arrows) and annulus (red arrows), which are ring‐like structures separating the midpiece and principal regions of the tail (Figure 2B; left panel). Spermatozoa from the affected individual A exhibited compound heterozygous mutations of *AGTPBP1* (NM_001286717.1: c.1336A > T [p.Glu423Asp] and c.1959C > T [p.Pro653Leu]), while affected individual B carried a hemizygous mutation of *AGTPBP1* (c.2499G > A [p. Arg811His]) that resulted in head defects (Figure 2B; middle panel) and lack of tail development (Figure 2B; left panel), respectively. Contrast immunostaining using sperm from the control, affected individual A, and affected individual B revealed mislocalized AGTPBP1 signals to the midpiece that had fragmented patterns (white arrows). Furthermore, spermatozoa from affected individual B showed significantly decreased AGTPBP1 signals (Figure 2B; Right panel). TEM revealed severe morphological and size abnormalities of the sperm head of spermatozoa from affected individual B (Figure 2C; red arrows). Most sperm showed no tail development, and a small portion of the sperm with tails showed disarranged mitochondria (Figure 2C; black arrows). These results indicated that genetically altered *AGTPBP1* expression resulted in spermatozoa with severe head and tail defects.

### 3.3 Dynamic patterns of AGTPBP1 during murine spermiogenesis

**FIGURE 3:**
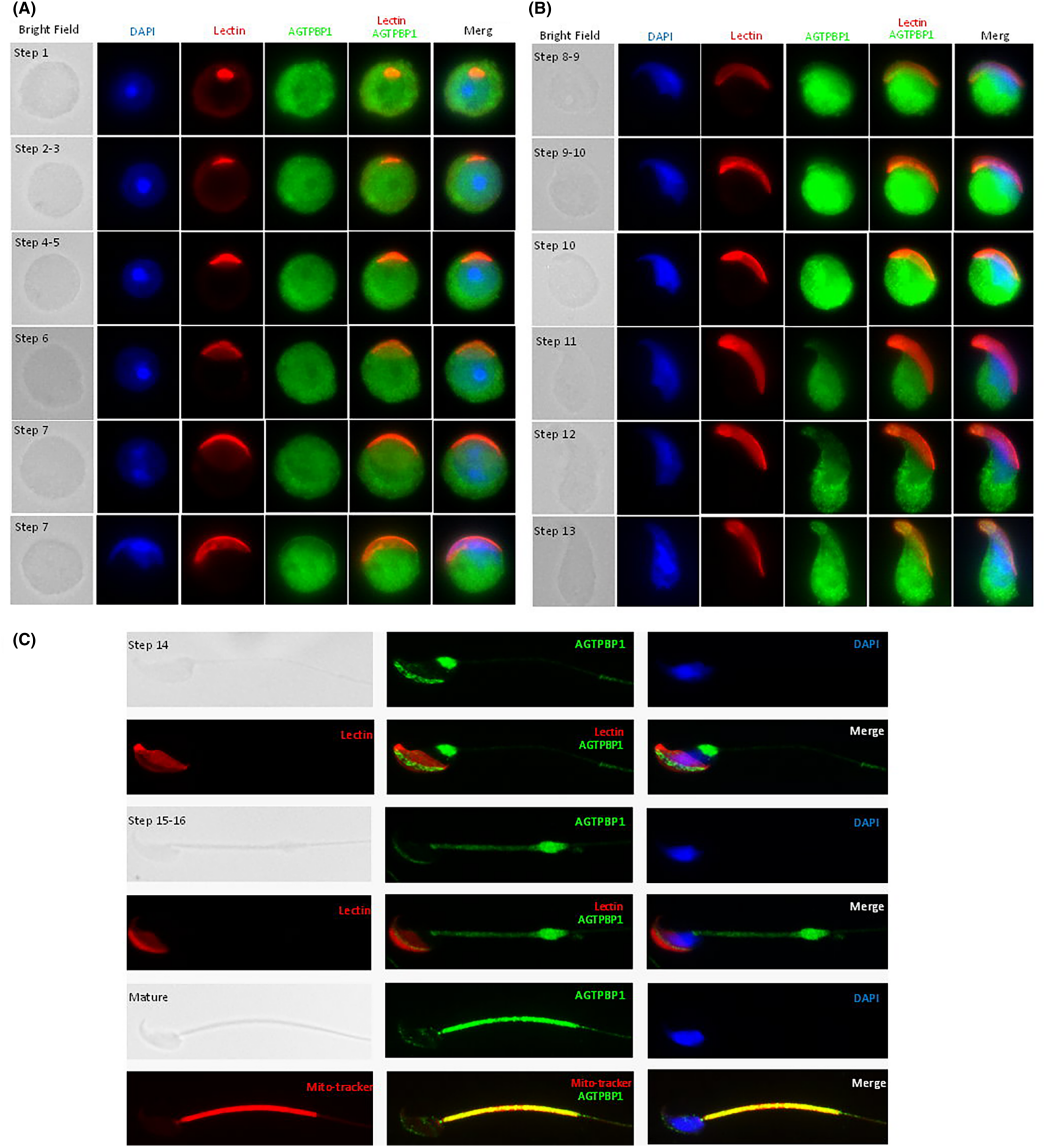
ATP/GTP Binding Protein 1 (AGTPBP1) signals showed multiple localizations during murine spermiogenesis. From left to right: bright field, DAPI staining (blue), lectin staining (acrosome marker; red), AGTPBP1 staining (green), AGTPBP1 staining (green) combined with lectin, and AGTPBP1 signals (lectin; AGTPBP1) combined with DAPI (Merge). (A–C) AGTPBP1 signals localized at different steps of murine spermiogenesis: step 1, step 2–3, step 4–5, Step 6, step 7, step 8–9, step 9–10, step 10, step 11, step 12, step 13, step 14, step 15–16, and mature sperm (co‐staining with Mito‐tracker). 400X magnification.

To determine the precise localization and possible reproductive role of AGTPBP1 during murine spermiogenesis, testicular germ cell populations were separated and subjected to immunostaining. In steps 1–7, round spermatids containing AGTPBP1 were distributed around the whole cells (Figure 3A). During shaping of the sperm head in steps 8–10, AGTPBP1 moved towards the post‐acrosomal region and was then recruited to the manchette structure in steps 11–13 (Figure 3B). Subsequently, AGTPBP1 was concentrated at the sperm neck (Step 14), followed by the removal of the middle piece at steps 15–16 **(**Figure 3C **)**. In mature caudal epididymal spermatozoa, AGTPBP1 was localized in the midpiece region (Figure 3C **).** These results suggest that AGTPBP1 is highly expressed during spermiogenesis and is likely involved in sperm head and tail formation.

### 3.4 Loss of *Agtpbp1* in mice causes severe morphological defects in the sperm

**FIGURE 4:**
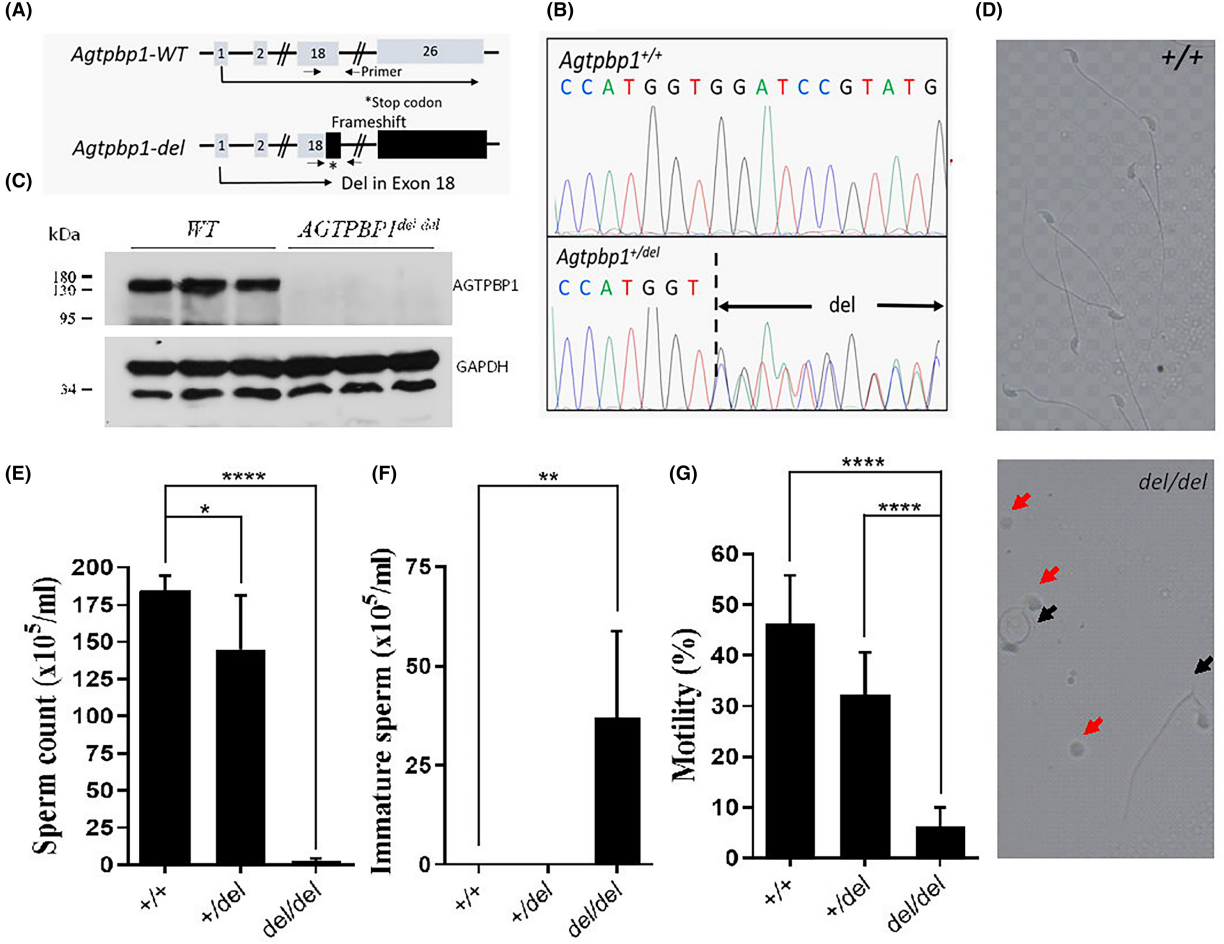
Disruption of *ATP/GTP Binding Protein 1* (*Agtpbp1*) allele in mice results in male sterility. (A) Schematic representation of the murine genomic structure and disrupted exon 18 of *Agtpbp1* induces frameshift mutation. (B) Sanger sequencing reveals the specific deletion (del) sites within exon 18 in *Agtpbp1* ^*+/del*^ mice, compared with wild‐type mice. (C) AGTPBP1 expression was evaluated in the murine testes of *Agtpbp1* ^*del/del*^ mice via western blotting. (D) Sperm collected from vas deferens of *Agtpbp1* ^*del/del*^ mice shows immature sperm (round‐like; red arrows) and defective sperm tail (black arrows), compared with the wild‐type mice (upper). (E–G.) Analysis of the ratio of sperm count, immature sperm (round‐like), and motility in 2‐month‐old wild‐type and *Agtpbp1* ^*del/del*^ mice. Mice number per genotype: wild‐type, *n* = 5; *Agtpbp1* ^*+/del*^, *n* = 5; *Agtpbp1* ^*del/del*^, *n* = 6. Sperm number > 200 per mouse. Each bar represents the mean ± standard error of the mean (SEM). *Significant differences (*p < 0.05; **p < 0.001; ****p < 0.00001, analysed using Student's *t* test).

To further test the causal relationship between genetic changes in *AGTPBP1* and male sterility, we generated an *Agtpbp1* ^*del*^ mouse line. (Figure 4A **)**. *AGTPBP1*‐deficient mice were confirmed by genotyping and immunoblotting (Figure 4B,C). *Agtpbp1* ^*del/del*^ mice were completely sterile when paired with controls (wild‐type male mice: 8 ± 0.82 pups per litter, *n* = 5 vs. *Agtpbp1* ^*del/del*^ male mice: 0 pups per litter, *n* = 6). Sperm isolated from the vas deferens and epididymis of *Agtpbp1* ^*del/del*^ male mice revealed severe abnormalities in sperm morphology and motility (wild‐type: *n* = 5; *Agtpbp1* ^*+/del*^: *n* = 5; *Agtpbp1* ^*del/del*^: *n* = 6) (Figure 4 D‐G). Notably, sperm morphology in *Agtpbp1* null mice displayed severe defects, such as immature sperm (no tail development) and tail defects (Figure 4D,F), which was similar to the results observed in *AGTPBP1‐*mutated patients **(**Figure 2B,C **)**. These results revealed that AGTPBP1 is required for normal sperm development and male fertility (Figure 4).

### 3.5 *Agtpbp1* deficiency affects the de‐polyglutamylation of tubulin during sperm development

**FIGURE 5:**
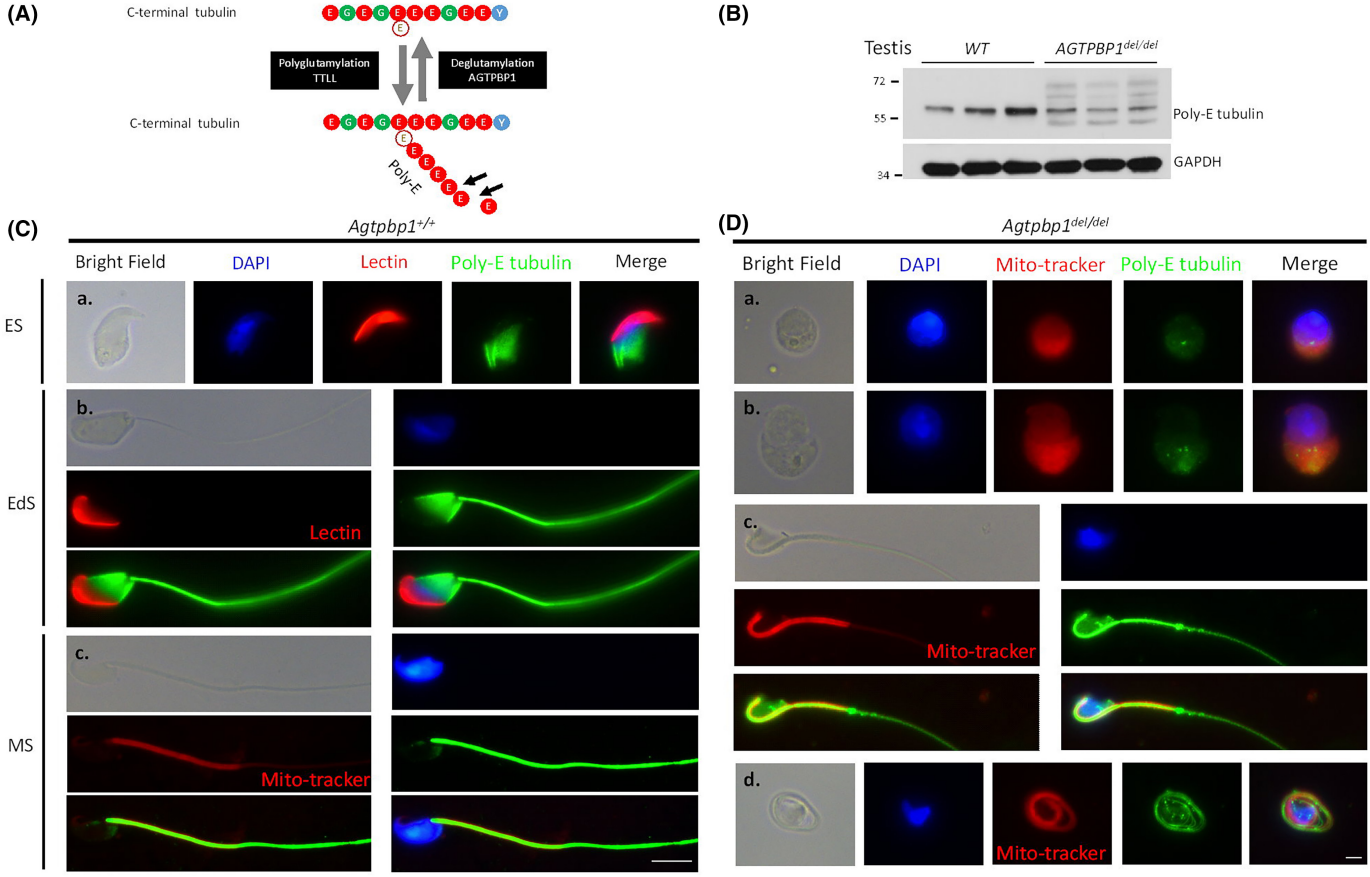
Loss of ATP/GTP Binding Protein 1 (AGTPBP1) affects its enzymatic function of depolyglutamation. (A) Diagrammatic representation of the tubulin tyrosine ligase‐like family protein (TTLL) and AGTPBP1 catalysing the polyglutamation and depolyglutamation of C‐terminal amino acids of tubulin, respectively. E: Glutamine; G: Glycine; Y: Tyrosine; poly‐E: Poly‐glutamination. (B) Amounts of polyglutamylated tubulin of various sizes were determined in the murine testicular tissue of *Agtpbp1* ^*del/del*^ mice (*n* = 3) via immunoblotting, compared with wild‐type (*n* = 3). (C) Different developmental steps of male germ cells and mature sperm isolated from wild‐type murine testis and vas deferens, respectively. (a). Elongating spermatids (ES); (b). elongated spermatids (EdS); and (c, d). mature sperm (MS). (D) Sperm isolated from vas deferens of *Agtpbp1* ^*del/del*^ mice. (C, D) From left to right: bright field, DAPI staining (blue), lectin staining (acrosome marker; red), anti‐polyglutamylated tubulin (poly‐E tubulin; green), and combined with DAPI, lectin, and poly‐E tubulin signals (Merge). Mature sperm co‐stained with Mito‐tracker (midpiece marker) (400X magnification).

The precise regulation and maintenance of tubulin stability are vital for the morphological formation of the sperm head and elongation of the tail, which are dependent on the stabilization of the microtubule structure.^22^ Precise regulation of the balance between polyglutamylation (poly‐E) and de‐polyglutamylation in the C‐terminal region of tubulin is mediated by TTLL and AGTPBP1^16^, ^31^ (Figure 5A). Therefore, we hypothesized that the loss of AGTPBP1 in mice results in an overabundance of polyglutamylated microtubules in developing germ cells and sperm. To test this hypothesis, we performed immunoblotting and immunostaining of *Agtpbp1*‐deficient testicular tissues to evaluate whether the loss of *Agtpbp1* affected the stability of poly‐E tubulin. Compared to wild‐type mice, the testicular tissues of *Agtpbp1*‐deficient mice showed abnormal poly‐E tubulin lengths **(**Figure 5B **).** Within germ cells from wild‐type mice, poly‐E tubulin was organized as a filamentous manchette structure in elongating spermatids and elongated spermatids during sperm head formation (Figure 5C‐a,b) Furthermore, poly‐E tubulin was present in the tails of mature sperm (Figure 5C‐c). In contrast, the loss of *Agtpbp1* in mice resulted in disorganized poly‐E tubulin in sperm with no tail development (Figure 5D‐a,b) or a defective tail (bent and curled) (Figure 5D‐c,d). Thus, the loss of *Agtpbp1* disrupts the stability of poly‐E‐tubulin, which is critical for sperm head and tail formation.

### 3.6 Loss of AGTPBP1 decreases the amount and disturbs the localization of △−2 tubulin during sperm head and tail formation

**FIGURE 6:**
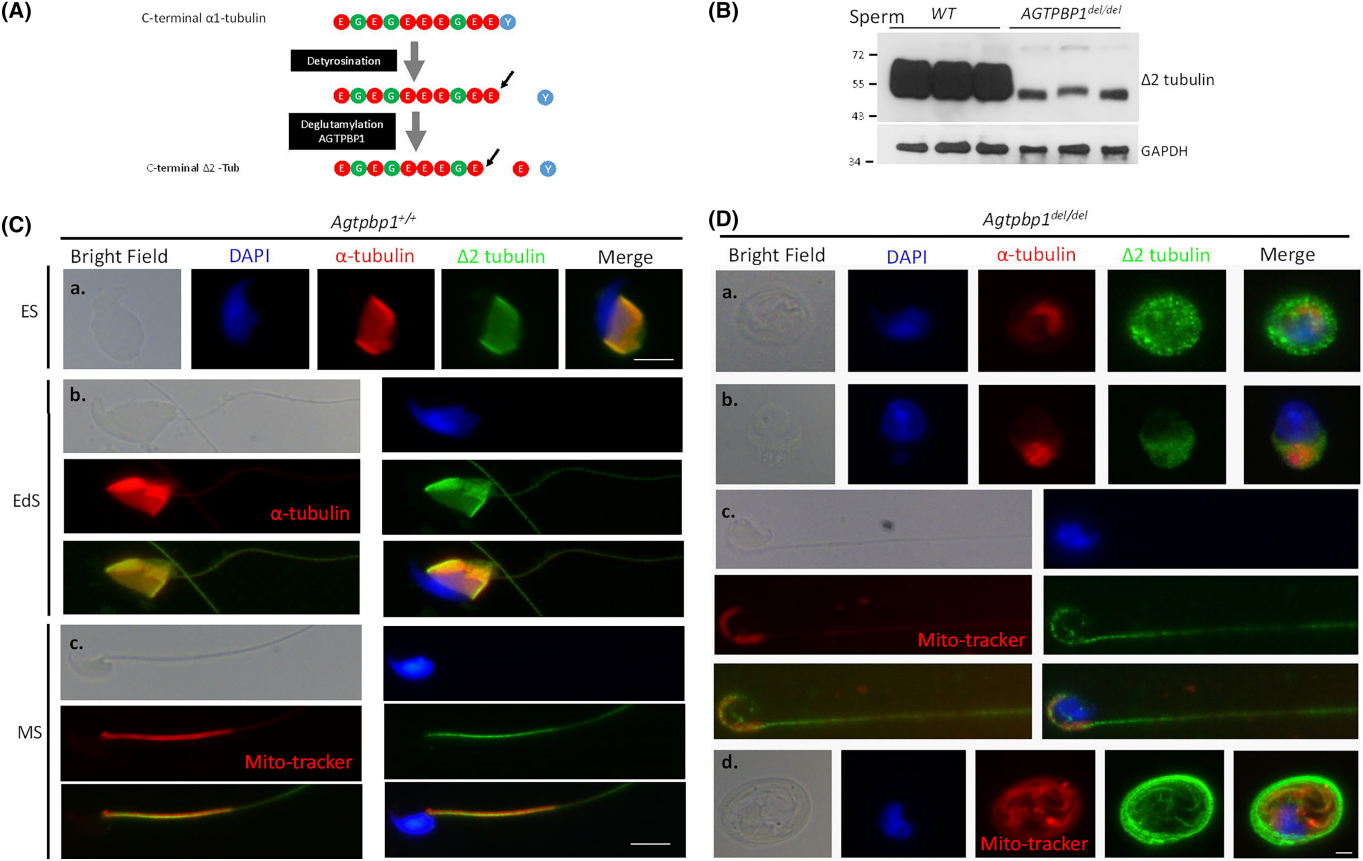
Deletion of *ATP/GTP Binding Protein 1 (AGTPBP1)* disrupts the generation of delta‐2 tubulin. (A) Elimination of polyglutamation (E) by AGTPBP1 C‐terminal amino acids of tubulin after tyrosine (Y) removal. (B) Amount of delta (△)‐2 tubulin analysed in the murine testis from wild‐type (*n* = 3) and *Agtpbp1* ^*del/del*^ mice (*n* = 3) using immunoblotting. (C) Different developmental stages of male germ cells and mature sperm isolated from wild‐type murine testes and vas deferens, respectively. a. Elongating spermatids (ES); b. elongated spermatids (EdS); and c. mature sperm (MS). (D) Sperm isolated from vas deferens of *Agtpbp1* ^*del/del*^ mice. (C, D) From left to right: bright field, DAPI staining (blue), α‐tubulin staining (red), anti‐△2 tubulin (△2 tubulin; green), and merged with DAPI, α‐tubulin, and anti‐△2 tubulin signals (Merge). Mature sperm co‐stained with Mito‐tracker (midpiece marker) (400X magnification).

The deglutamylation activity of AGTPBP1 is a critical step for △−2 tubulin generation (Figure 6A). Therefore, we investigated whether the disrupted *Agtpbp1* allele affected the generation of △−2 tubulin. Figure 6B shows a significant decrease in the amount of △−2 tubulin in the *Agtpbp1* ^*del/del*^ testis, compared with that in wild‐type mice. In wild‐type mice, △−2 tubulin is a major component of the manchette (Figure 6C‐a,b) and sperm tail (Figure 6C‐c) at various developmental stages: elongating spermatids, elongated spermatids, and mature sperm. In contrast, the loss of AGTPBP1 function resulted in a disrupted △−2 tubulin structure within the sperm with no tail development (Figure 6D‐a,b) and morphologically defective sperm (Figure 6D‐c,d). Collectively, these data indicate that AGTPBP1 plays an essential role in the generation of △−2 tubulin in developing male germ cells (Figure 6).

## 4 DISCUSSION

In this study, three sporadic genetic alterations in *AGTPBP1* in Taiwanese patients with teratozoospermia were identified using WES. Sperm from affected patients showed severe structural head and tail defects. AGTPBP1 was mainly observed during murine sperm head formation and tail elongation during spermiogenesis. Consistent with these findings, sperm from a *Agtpbp1* deletion mouse model exhibited morphological defects in the head and tail. At the molecular level, the loss of *Agtpbp1* resulted in an abnormal ploy‐E tubulin length and decreased △−2 tubulin generation in vivo. To the best of our knowledge, this is the first study to link genetic changes in *AGTPBP1* with teratozoospermia from a clinical perspective.

### 4.1 Identification of male sterility–related genetic changes in Taiwan from WES

Several studies investigating sterility‐related genetic alterations have been conducted; however, these genetic alterations differ among populations. In this study, deleterious genetic changes were identified in seven genes using WES and data obtained from PSAP, ExAC, PolyPhen, and SIFT analyses. Five of these genes were characterized as male infertility‐related genes, including *Polo‐like kinase 4* (*PLK4*)*, AGTPBP1, KISS1 receptor*, *Meiosis Expressed Gene 1* (*MEIG1*), and *Piwi Like RNA‐Mediated Gene Silencing 2* (*PIWIL2*) **(**Figure 1 **)**. PLK4, which belongs to the polo protein family of serine/threonine protein kinases,^32^, ^33^ is located in centrioles, which are microtubule‐based structures within centrosomes, and is involved in centriole formation.^33^ In clinical observations, a heterozygous 13‐bp deletion in the serine/threonine kinase domain of *PLK4* was identified in azoospermia cases with Sertoli cell‐only syndrome (SCOS),^34^ and a heterozygous *PLK4* mutation (p.Ile242Asn) in mice caused male germ cell loss in the testes,^35^ similar to that in human SCOS. Another gene, *MEIG1*, is involved in meiosis.^36^ *Meig1* knockout in male mice results in sterility because of the arrest of spermiogenesis before the completion of spermatid elongation.^37^ TEM revealed that the manchette structure was disrupted in spermatids of *Meig1*‐deficient mice. Based on previous reports, the mutated genes identified using WES in this study appear to be potential causative candidates for male infertility in Taiwan.

### 4.2 *AGTPBP1* mutations cause CONDCA

CONDCA has recently been identified as a rare and severe autosomal recessive disorder that affects neurodevelopment in the central and peripheral nervous systems.^19^ Shashi et al. (2018) identified CONDCA‐related deletion (del exon 1–12), splicing‐site mutation (c.2336‐1G > T), missense mutations (p.Y694D, p.R799C, p.T851M, p.R910C, p.R918W, and p.H990L), and nonsense mutations (p.R330*, p.Q788*, p.Q856*, and p.Y912*) in *AGTPBP1* using WES in 13 cases from 10 families.^19^ Furthermore, different CONDCA‐related mutations (p.R374*, p.R759L, p.T784C, p.R799L, and p.R1000*) and alteration in the splicing site (c.2342 + 2 T > G) of *AGTPBP1* have been characterized in different populations.^32^, ^38^, ^39^ In these studies, mutations affecting the amount of protein or the molecular functions of *AGTPBP1* were also analysed. The fibroblast cells collected from CONDCA cases with the mutants were found to have decreased *AGTPBP1* levels.^19^ Muscle tissues from patients with p.Q856* showed an accumulation of abnormally sized poly‐E tubulin. Moreover, HEK293 muscle cells transfected with the mutant *AGTPBP1* exhibited impaired deglutamylase activity, which decreased the abundance of the stable form of tubulin, △−2 tubulin. However, because CONDCA is a severe childhood‐onset neurodegenerative disease, its reproductive phenotype has not been evaluated. In the present study, we identified three genetic alterations in *AGTPBP1* in patients with teratozoospermia (affected individual A: p.Glu423Asp and p.Pro653Leu; affected individual B: p.Arg811His) (Figure 1). Spermatozoa from patients with *AGTPBP1* mutations exhibited disrupted patterns and decreased signals of AGTPBP1 as well as head and tail defects **(**Figure 2 **)**; however, neither patient showed neurodegenerative phenotypes (Figure 1). This suggests that different mutation sites in AGTPBP1 result in distinct genetic outcomes in neurons and male germ cells. Further investigation is required to understand the genetic changes in AGTPBP1 that lead to CONDCA and teratozoospermia.

### 4.3 Loss of the *Agtpbp1* allele in mice also causes male sterility

The recessive and spontaneous mouse mutation *Purkinje cell degeneration* (*pcd*) causes early onset degeneration of the cerebellar Purkinje cells, retinal photoreceptor cells, and olfactory bulb and thalamic neurons.^40^, ^41^ In addition to neuronal degeneration, *pcd* mutant male mice also exhibit male sterility, with decreased sperm counts and few mature sperm.^32^ After genetic mapping, the mutated gene of the *pcd* strain mice was mapped to chromosome 13, and mutations, deletions, and insertions of genomic fragments within the *AGTPBP1* gene were identified in *pcd* ^*2J*^ *, pcd* ^*3J*^, and *pcd* ^*5J*^ strains,^20^, ^42^ respectively. The mRNA expression levels of *AGTPBP1* were found to be reduced in the brain and testis tissues of *pcd* mice.^20^, ^42^ Kim et al. (2011) first detected AGTPBP1 in spermatocytes and found it to be primarily expressed in round spermatids in testicular sections.^21^ Loss of the *AGTPBP1* allele in *pcd* ^*3J*^ mice decreased testicular weight, increased apoptotic cell death in the testes, and produced abnormally shaped spermatozoa from the cauda epididymis.^37^ However, the detailed dynamic expression patterns and in vivo functions of *AGTPBP1* during sperm morphological development remain unclear. In this study, we found that *AGTPBP1* was specifically localized around the manchette structure of the sperm head and elongating tail during murine spermiogenesis **(**Figure 3 **)**. Significantly reduced sperm count and motility and an increased number of immature (no tail development) and tail‐defective sperm were observed in our *Agtpbp1‐*knockout mice **(**Figure 4 **)**. Immature sperm are released from the seminiferous tubules owing to disrupted sperm development. These findings indicate that AGTPBP1 is critical for sperm head and tail formation during murine spermatogenesis.

### 4.4 Deglutamylation activity of AGTPBP1 is critical for the formation of sperm head and tail in mice

The balance between polyglutamylation and deglutamylation in the C‐terminus of tubulin is critical for the stability of the microtubule structure during neuronal development.^43^ Polyglutamylases and deglutamylation modifications are catalysed by tubulin tyrosine ligase‐like proteins, TTLL family proteins, and cytosolic carboxypeptidase family proteins (e.g., AGTPBP1).^44^, ^45^ Using *pcd* strain, Shashe et al. reported that the femoral quadriceps nerve of *pcd* ^*3J*^ mice exhibited a reduction in nerve diameter and the number of myelinated axons formed via microtubule polymerization.^19^ Furthermore, the cerebellum of *pcd* ^*3J*^ mice exhibited the increased tubulin polyglutamylation and decreased △−2 tubulin generation. These results support the hypothesis that *AGTPBP1* is critical for maintaining microtubule structure, the major cytoskeletal component of the axon, and preserves neuron numbers. In this study, *Agtpbp1* ablation in mice revealed similar results: an increase in abnormally sized polyglutamylated tubulin and decrease in △−2 tubulin levels (Figure 5B and 6B). Compared with signals from wild‐type mice, the multiplex sizes of polyglutamylated tubulin and △−2 tubulin exhibited fragmented patterns during sperm head and tail formation (Figure 5C and 6C). Therefore, we suggest that the deglutamylation activity of AGTPBP1 is critical for the formation and maintenance of neurons and male germ cells.

### 4.5 Enzymatic and molecular roles of AGTPBP1 during murine spermiogenesis

**FIGURE 7:**
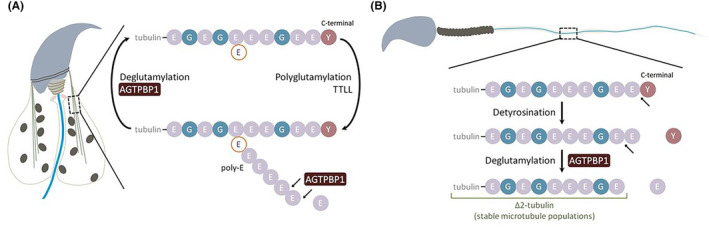
Possible enzymatic and molecular roles of ATP/GTP Binding Protein 1 (AGTPBP1) in sperm head and tail formation. During the elongation of the sperm head and tail, AGTPBP1 is highly localized at the manchette (A) and axoneme (B). (A) The polyglutamylation and depolyglutamylation of C‐terminal sequences of tubulin were catalysed by tubulin tyrosine ligase‐like family proteins (TTLL) and AGTPBP1, respectively, to balance the long chain of polyglutamylation in tubulin. (B) AGTPBP1 also catalyses the generation of △2‐tubulin (stable microtubules), which requires prior detyrosination. E, glutamine; G, glycine; Y, tyrosine; poly‐E, poly‐glutamination.

Based on our results and those of previous studies, we proposed a possible molecular model of AGTPBP1 during spermiogenesis **(**Figure 7 **)**. AGTPBP1 was highly localized in the manchette structure in the heads of elongating spermatids and the tails of mature sperm **(**Figure 3 **)**. The manchette, a temporary microtubule and an actin‐based structure, facilitates the transport of vesicles and proteins necessary for the formation of the sperm head and tail.^22^, ^46^ Figure 7A shows that polyglutamylation and depolyglutamylation of the C‐terminal sequences of tubulin are catalysed by TTLL and AGTPBP1, respectively, to balance long‐chain polyglutamylation in tubulin. AGTPBP1 also catalyses the generation of △2‐tubulin to form stable microtubulin after the detyrosination of tubulin **(**Figure 7B **)**. In *AGTPBP1*‐defective mice, the manchette and sperm tail structures were disrupted **(**Figures 5 and 6 **)** due to the impairment of the enzymatic functions of AGTPBP1.

The present study is the first to establish a connection between the genetic mutations in *AGTPBP1* to teratozoospermia. Damage to *AGTPBP1* in human and mouse sperm results in severe sperm tail and head defects, leading to the loss of deglutamylation function. This study identifies the role of *AGTPBP1* mutations in teratozoospermia and provides potential guidance for the diagnosis and treatment of male infertility.

## AUTHOR CONTRIBUTIONS

**Yu‐Hua Lin:** Conceptualization (equal); data curation (equal); funding acquisition (equal). **Ya‐Yun Wang:** Data curation (equal); investigation (equal); project administration (equal). **Tsung‐Hsuan Lai:** Data curation (equal); methodology (equal); resources (equal). **Jih‐Lung Teng:** Investigation (equal); methodology (equal); software (equal). **Chi‐Wei Lin:** Investigation (equal); software (equal). **Chih‐Chun Ke:** Funding acquisition (equal); investigation (equal). **I‐Shing Yu:** Investigation (equal); methodology (equal). **Hui‐Ling Lee:** Investigation (equal); methodology (equal). **Chying‐Chyuan Chan:** Investigation (equal); resources (equal). **Chi‐Hua Tung:** Software (equal); visualization (equal). **Donald F. Conrad:** Formal analysis (equal); methodology (equal); software (equal). **Moira K. O’Bryan:** Conceptualization (equal); writing – original draft (equal); writing – review and editing (equal). **Ying‐Hung Lin:** Conceptualization (equal); funding acquisition (lead); writing – original draft (equal).

## CONFLICT OF INTEREST STATEMENT

The authors declare that they have no conflicts of interest.

## Supporting information


**Table S1:** Clinical features of the 12 men with teratozoospermia

## Data Availability

The data that support the findings of this study are available on request from the corresponding author. The data are not publicly available due to privacy or ethical restrictions.
