# Supplementary figures and images for: Deleterious genetic changes in AGTPBP1 result in teratozoospermia with sperm head and flagella defects
Source: J Cell Mol Med. 2023 Nov 8;28(2):e18031. doi: 10.1111/jcmm.18031 (PMC10826451; doi:10.1111/jcmm.18031)

**Supplemental Data**

**Table. Clinical features of the 12 men with teratozoospermia**


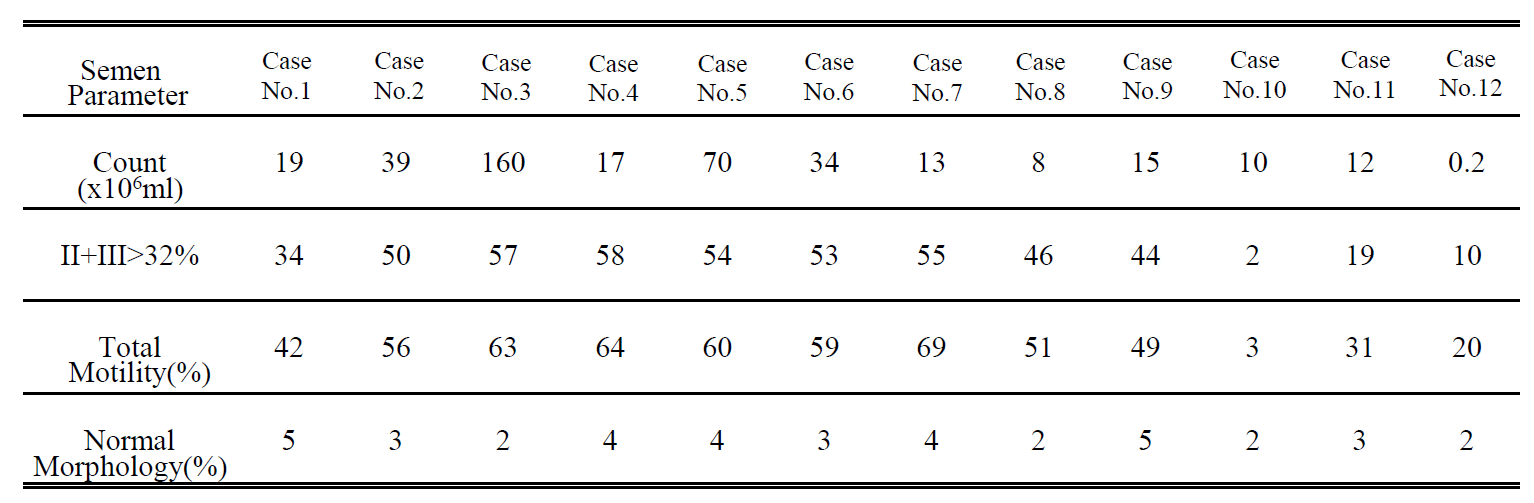

Supplement: Supplementary file 1 — Table S1: Clinical features of the 12 men with teratozoospermia [file JCMM-28-e18031-s001.docx]
